# Phytophotodermatitis

**Published:** 2013-09-02

**Authors:** Karim A. Sarhane, Amir Ibrahim, Shawn P. Fagan, Jeremy Goverman

**Affiliations:** ^a^Department of Plastic and Reconstructive Surgery, Johns Hopkins University School of Medicine, Baltimore, Md; ^b^Massachusetts General Hospital, Division of Burns and Shriners Hospital, Boston, Mass.

**Keywords:** allergic contact dermatitis, burn, chemical burn, limes, phytophotodermatitis

**Figure:**
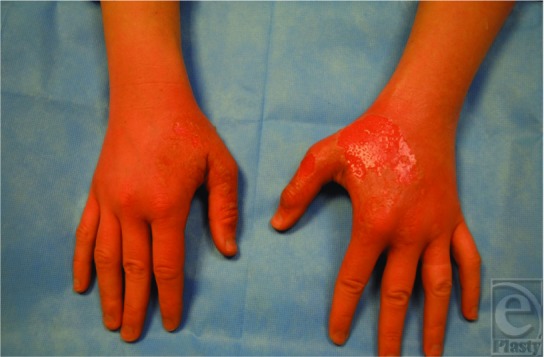


## DESCRIPTION

A 30-year-old woman presented to the emergency department with a 2-day history of painful blistering wounds over the dorsum of both hands. She reported her hands “began to peel” 24 hours after she had sliced limes for a beach party; no other precipitating events (chemical or thermal trauma) were mentioned.

## QUESTIONS

1. **What is the diagnosis?**
2. **What is the mechanism of injury?**
3. **How would you treat this patient?**
4. **What are the long-term sequelae of this injury?**

## DISCUSSION

A diagnosis of phytophotodermatitis was made. Tender erythematous patches studded with blisters were noted over the dorsum of both hands.

Phytophotodermatitis is a nonimmunologic phototoxic cutaneous eruption resulting from contact with photosensitizing substances found in plants; furocoumarins (present in limes and other plants) are typically implicated and get activated following exposure to sunlight (especially ultraviolet A rays, 320-400 nm).^1^ Although simple photoallergic reactions may occur, phototoxicity is more common. Two types of toxic reactions occur: one is oxygen independent, in which the ultraviolet-activated furocoumarins bind to RNA and nuclear DNA; another is oxygen dependent, where induced furocoumarins cause cell membrane damage and edema.^2^^,^^3^ These reactions ultimately lead to cell death (sunburned cells and apoptotic keratinocytes). Burning erythema, blistering rash, and oftentimes tense bullae appear in the subsequent 24 hours, and peak around 48 to 72 hours. Wet skin, sweating, and heat all enhance this phototoxic response.^4^ In some cases of oral intake of phototoxic plants, severe skin inflammation and necrosis might occur in areas exposed to sunlight.^5^ Diagnosis is occasionally difficult because erythema and vesicles in phytophotodermatitis may mimic atopic dermatitis, type IV hypersensitivity reaction, or chemical burns.^6^

Treatment of phytophotodermatitis depends on its extent of involvement. In mild cases, conservative management with a moist dressing is acceptable. In severe cases or in those involving more than 30% of total body surface area, admission to a burn unit is recommended for local wound care. Cooling the acute lesions and topical corticosteroid application may help to alleviate patient discomfort.^7^ Systemic treatment with corticosteroid is advocated in extremely severe cases of skin inflammation with necrosis. In the patient presented here, conservative management with daily bacitracin and dry sterile dressing was used along with frequent hand exercises to prevent stiffness.

Hyperpigmentation often develops 1 to 2 weeks after epithelialization and may last for many months before fading. In most scenarios, it is a psoralen (a furocoumarin)-induced hyperpigmentation; it occurs through increased melanocyte mitosis and dendricity, melanocyte hypertrophy, increased tyrosinase activity, and changes in the size and distribution of melanosomes.^4^ Avoiding sunlight and photosensitizing agents is strongly recommended after the initial acute reaction. In some cases, however, namely in phytophotodermatitis resulting from contact with fig trees, hypomelanosis instead develops; the underlying mechanism is less clear, but it is assumed to involve apoptosis of melanocytes.^8^

In summary, phytophotodermatitis may be induced by skin contact with limes followed by ultraviolet light exposure. Skin lesions have sharp demarcations (like in our patient); burning sensations and pain are conspicuous. Diagnosis is challenging, and confusion with other skin conditions may delay treatment. In this regard, health care professionals need to be alert to this condition. To prevent severe injuries from this entity, public education is essential about all possible causes and manifestations of phytophotodermatitis.
